# Candidate Markers Associated with the Probability of Future Pulmonary Exacerbations in Cystic Fibrosis Patients

**DOI:** 10.1371/journal.pone.0088567

**Published:** 2014-02-12

**Authors:** Gabriella Wojewodka, Juan B. De Sanctis, Joanie Bernier, Julie Bérubé, Heather G. Ahlgren, Jim Gruber, Jennifer Landry, Larry C. Lands, Dao Nguyen, Simon Rousseau, Andrea Benedetti, Elias Matouk, Danuta Radzioch

**Affiliations:** 1 Department of Human Genetics, McGill University, Montreal, Quebec, Canada; 2 Institute of Immunology, Central University of Venezuela, Caracas, Venezuela; 3 Adult Cystic Fibrosis Clinic, Montreal Chest Institute, McGill University Health Center, Montreal, Quebec, Canada; 4 McGill University Health Center Research Institute, Montreal, Quebec, Canada; 5 Department of Medicine, McGill University, Montreal, Quebec, Canada; 6 Division of Pediatric Respiratory Medicine, Montreal Children's Hospital, McGill University Health Center Research Institute, Montreal, Quebec, Canada; 7 Department of Epidemiology, Biostatistics and Occupational Health, Montreal Chest Institute, McGill University Health Center, Montreal, Quebec, Canada; University of Duisburg-Essen, Germany

## Abstract

**Introduction:**

Pulmonary exacerbations (PEs) cause significant morbidity and can severely impact disease progression in cystic fibrosis (CF) lung disease, especially in patients who suffer from recurrent PEs. The assessments able to predict a future PE or a recurrent PE are limited. We hypothesized that combining clinical, molecular and patient reported data could identify patients who are at risk of PE.

**Methods:**

We prospectively followed a cohort of 53 adult CF patients for 24 months. Baseline values for spirometry, clinical status using the Matouk Disease Score, quality of life (QOL), inflammatory markers (C-reactive protein (CRP), interleukins (IL)-1β, -6, -8, -10, macrophage inflammatory protein (MIP)-1β, tumor necrosis factor (TNF) and vascular endothelial growth factor (VEGF)), polyunsaturated fatty acids and lipid peroxidation in blood plasma were collected for all patients during periods of stable disease, and patients were monitored for PE requiring PO/IV antibiotic treatment. Additionally, we closely followed 13 patients during PEs collecting longitudinal data on changes in markers from baseline values. We assessed whether any markers were predictors of future PE at baseline and after antibiotic treatment.

**Results:**

Out of 53 patients, 37 experienced PEs during our study period. At baseline, we found that low lung function, clinical scoring and QOL values were associated with increased risk of PE events. PEs were associated with increased inflammatory markers at Day 1, and these biomarkers improved with treatment. The imbalance in arachidonic acid and docosahexaenoic acid levels improved with treatment which coincided with reductions in lipid peroxidation. High levels of inflammatory markers CRP and IL-8 were associated with an early re-exacerbation.

**Conclusion:**

Our results demonstrate that worse clinical and QOL assessments during stable disease are potential markers associated with a higher risk of future PEs, while higher levels of inflammatory markers at the end of antibiotic treatment may be associated with early re-exacerbation.

## Introduction

Cystic fibrosis (CF) patients often suffer acute exacerbations of their pulmonary symptoms, necessitating more aggressive treatment. Pulmonary exacerbations (PEs) are major events contributing to the morbidity and progression of CF lung disease. The recovery from PEs is largely based on the reversal of symptoms and improvement in lung function. However, pre-PE lung function levels are not recovered in 15% to 25% of CF patients[1], [2]. Even when pulmonary function tests return to normal values, experiencing a PE contributes to long-term decline in lung disease with similar impacts on survival as would a 12% reduction in lung function[3], [4]. Higher frequencies of PEs were associated with greater rates of decline in forced expiratory volume in one second percent predicted (FEV_1_%), especially having more than two PEs per year could increase the need for transplant and the risk of death[5]. The survival model characterized by Liou et al. also predicted that the impact on 5-year mortality of four PE events in a single year was as detrimental as *Bulkholderia cepacia* infection or a 48% reduction in FEV_1_%[3]. Given the substantial morbidity and mortality associated with PEs, there is an urgent need to identify patients at risk of PE, particularly recurrent PEs. Improving the clinicians' ability to stratify patients based on their risk to develop PEs will allow for more effective prevention (eg. treatment of CF related diabetes or allergic bronchopulmonary aspergillosis) and early intervention to prevent irreversible lung damage. Although standards of care that include inhaled antibiotics, azithromycin, recombinant human deoxyribonuclease and hypertonic saline contribute in reducing the frequency of exacerbations, there is currently no effective and reproducible diagnostic marker for the identification of early stages of PE. The standard criteria used to monitor PEs are mainly focused on lung function indicators such as FEV_1_% which are mostly reflective of disease severity and not necessarily disease activity[6]. Patient reported symptoms are important complements to physician-documented clinical signs in the diagnosis of PE. In fact, newly developed diaries are being validated for the purpose of early intervention to quickly reduce the development of a full and vigorous inflammatory response and to shorten and reduce the severity of PE with the hope of preventing the development of irreversible lung damage[7].

The events that trigger PEs are still poorly understood and may include respiratory viral infections[8], [9] and air pollution[10]. Bacterial pathogens already present in the patients' lungs may be causing PEs by adapting their virulence or colonizing new areas of the lung[11]. Retrospective studies have found risk factors for developing PEs which include the female sex, nutritional status, CF co-morbidities such as CF related diabetes, pancreatic insufficiency, and lung microbiology (eg. *Aspergillus fumigatus, Bulkholderia cepacia*)[2], [3], [5], [12], [13]. Due to the nature of data available to perform retrospective analyses, these risk factors are based on clinical information which help to identify a population of CF patients more likely to have PEs. However on an individual basis, it remains difficult to assess which patients will experience a PE.

There have been few prospective studies looking at factors associated with risk of PEs which included molecular markers as predictors. A study by Sequeiros et al. found that the time to the next PE was shorter in patients with allergic bronchopulmonary aspergillosis and CF related diabetes. The authors also described that high C-reactive protein (CRP) and low FEV_1_ values at the end of antibiotic PE treatment were associated with shorter times until the next PE. They did not find any other molecular markers of significance[14]. Gray et al. demonstrated that serum calprotectin levels at the end of PE treatment rather than CRP were linked to the time until next PE[15]. These two studies show the potential of inflammatory markers in predicting risk of future PE.

For this study, we asked two questions: 1) In a period of stable disease, who is more likely to have a PE in the future? and 2) After PE treatment, who is at risk of an early re-exacerbation? We hypothesized that combining clinical and patient reported data with inflammatory markers and fatty acids may result in a better evaluation of patient disease activity. More specifically, we describe that worse disease-specific patient reported quality of life (QOL) and clinical assessments during stable disease indicated risk of PE, while higher levels of inflammation at the end of PE treatment were associated with early re-exacerbation.

## Materials and Methods

### Study design

#### Primary study: Markers at stable disease associated with risk of future PE

CF patients from the Adult Cystic Fibrosis Clinic at the Montreal Chest Institute (Montreal, Qc, Canada) were approached during their regular clinic visit to participate in the study. Fifty-three patients were enrolled in our prospective cohort study and were followed for a 12 month period from enrollment. The study duration was 24 months in total. Baseline data was recorded during a period of stable disease defined by the absence of any PE requiring intravenous (IV) or oral antibiotic therapy in the preceding month. One patient was excluded from the study after baseline data was collected due to lung transplantation, thus we had 52 patients in our patient group. Inhaled antibiotics (tobramycin or aztreonam) were prescribed as maintenance therapy to 39 patients enrolled in the study (75% of the cohort: 32 patients that had PE and 7 that had no PE). A PE was defined as any change in patients' symptoms (increased cough, sputum production and breathlessness, and decreases in lung function, weight, appetite and energy) requiring additional oral or IV antibiotic therapy [5]. The decision to treat was at the physicians' discretion and was not influenced by this study. All patients were treated with β-lactams and/or fluoroquinolones in addition to tobramycin. Some received additional antibiotics such as doxycycline, clindamycin or trimethoprim. Nine patients were given corticosteroids during their first PE of the study based on previous response and/or severe bronchospasm.

#### Secondary study: Markers at the end of PE treatment associated with early re-exacerbation

Among patients that experienced PE (n = 37), we collected serial longitudinal clinical data and blood samples throughout the PEs of 13 randomly selected patients. Data and samples were collected at Day 1 (within 24 hours prior to PE treatment, n = 13), and on follow-up assessments on Days 7 (n = 12), 14 (n = 11), 21 (n = 9) and 42 (n = 8). Four patients received treatment for 14 days and nine for 21 days. Patient compliance limited the sampling of all patients for Days 7, 14 and 21 time points. By Day 42, four patients had already re-exacerbated and were not included in this follow-up time point.

Volunteers serving as healthy controls (HC) were recruited from the McGill University Health Centre. CF patients and healthy controls gave written consent to participate in the study which was approved by the Institutional Review Board of the McGill University Health Centre.

### Clinical data and clinical scoring

At enrollment, patients' information on age, sex, weight and body mass index (BMI) was recorded. At baseline (n = 52) and at all defined time points during PEs (n = 8–13), the patients' weight, BMI, complete blood cell counts and clinical information for the Matouk Disease Score were assessed. The Matouk Disease Score (Matouk Modified N. Huang Disease Score), previously described and validated[16], [17], was used to quantify disease activity of CF patients. Briefly, the total score comprises of four subscores: Clinical (weight, weight change, dyspnoea, cough, sputum, physical exam, respiratory rate/breathing pattern/cardiac frequency, bacterial culture, appetite and general condition), Pulmonary Function (PFT: forced vital capacity percent predicted (FVC%), FEV_1_% predicted, FEV_1_/FVC, forced expiratory flow (FEF)_25–75%_ predicted, FEF_50%_ predicted, RV/TLC), Chest Radiography (CXR) score (based on the Bradsfield method for radiography scoring, comprised of scores for air trapping, linear markings, nodular cystic lesions, parenchymal lesions, general impression) and Complications score (number of previous PEs, pneumothorax, hemoptysis, respiratory failure, cardiac enlargement, pulmonary surgery). Healthier patients have higher scores for Clinical, PFT and CXR categories and lower values for the Complications score. To calculate the total score: Total score  =  Clinical+PFT+CXR−Complications. A full description of the scoring method can be found in File S1.

### Measures of lung function

At baseline (n = 52) and at all defined time points during PEs (n = 8–13), lung function was assessed by spirometry, and included FEV_1_% and FVC%. Spirometry was performed according to the American Thoracic Society standards[18], [19].

### Patient reported quality of life (QOL)

The QOL evaluation was recorded at baseline (n = 52) and at defined time points during PE (n = 8–13) by a self-administered questionnaire using the CF Questionnaire-Revised (CFQ-R) which comprised of 50 items associated with 3 symptom scales (Weight, Respiratory Function and Digestion) and 9 QOL domains (Physical, Vitality, Emotional, Eating Disturbances, Treatment Burden, Health Perceptions, Body Image, Social Functioning and Role/School Functioning) [20]. Higher scores reflect healthier disease status. The total score is the sum of scores for all items in the questionnaire.

### Plasma inflammatory biomarker analysis

At baseline (n = 52) and each defined time points during PEs (n = 8–13), blood samples were collected in ethylenediaminetetraacetic acid (EDTA) coated tubes and spun at 3000 rpm for 10 min at 4°C for plasma isolation. Plasma CRP levels were quantified in the hospital's clinical laboratory using ELISA. Cytokines were measured in plasma with the MILLIPLEX® Map multiplex assay kit (Millipore, Mississauga, ON, Canada) and the MAGPIX® multiplex system (Millipore) according to the manufacturer's instructions. The data was assessed for interleukin (IL)-1β, IL-6, IL-8, IL-10, macrophage inflammatory protein 1β (MIP-1β, also known as CCL4), tumor necrosis factor (TNF) and vascular endothelial growth factor (VEGF) using the MILLIPLEX® Analyst software, version 4.2 (Millipore). The concentrations of cytokines are expressed as pg/ml.

### Plasma polyunsaturated fatty acid and lipid peroxidation analysis

After plasma isolation as described above, 100 µl of plasma was added to 900 µl 2∶1 chloroform/methanol solution with added 1 mM of butylated hydroxyanisole (BHA) to prevent oxidation of the fatty acids. Samples were stored in −80°C until analysis. Lipids were isolated using the method described by Folch[21]. The polyunsaturated fatty acids (PUFA) in this fraction were esterified as described by Schlenk and Gellerman[22] and the esters were identified by gas chromatography/mass spectrometry (Hewlett Packard 5880A, WCOT capillary column (Supelco-10, 35 m×0.5 mm, 1 µm thick)) using commercial standards (Sigma-Aldrich, Oakville, ON, Canada)[23]. In addition, the total protein content of the aqueous phase was analysed using the bicinchoninic assay (Pierce Biotechnology, Rockford, IL, USA). The concentrations of arachidonic acid (AA) and docosahexaenoic acid (DHA) are expressed as nmol/µg of protein. The AA/DHA ratio represents the amount of AA to DHA in each patient. The AA/DHA ratio represents the amount of AA to DHA in each sample. Lipid peroxidation was assessed by indirectly measuring malondialdehyde (MDA) using the thiobarbituric acid reactive species (TBARS) assay[23] and is expressed as nmol of TBARS/mg of protein.

### Statistical analysis

**Figure 1:**
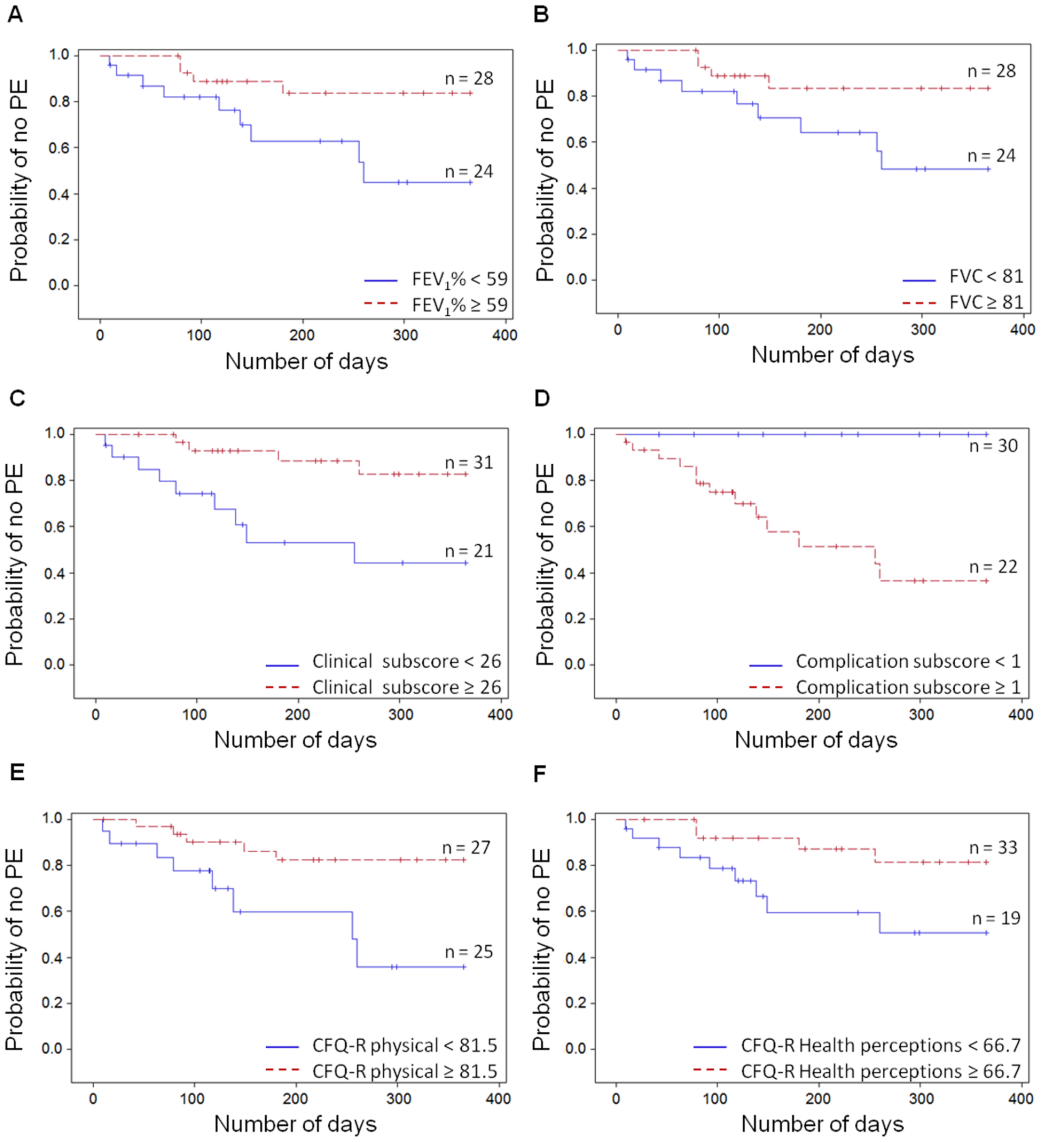
Probability of having a PE is associated with low clinical and QOL assessments. Using the Kaplan-Meier survival analysis, we evaluated whether the markers were associated with the risk of a PE. Continuous covariates were dichotomized at the median. The parameters illustrated here were all influencing the probability of having a PE. Lower risks of PE were associated with A) higher FEV1% predicted (p = 0.020), B) higher FVC% predicted (p = 0.032), C) higher Clinical subscore of the Matouk Disease Score (p = 0.004), D) lower Complications subscore of the Matouk Disease Score (p = 0.000), E) higher assessments of QOL physical (p = 0.030) and F) higher health perceptions (p = 0.006) domains.

**Figure 2:**
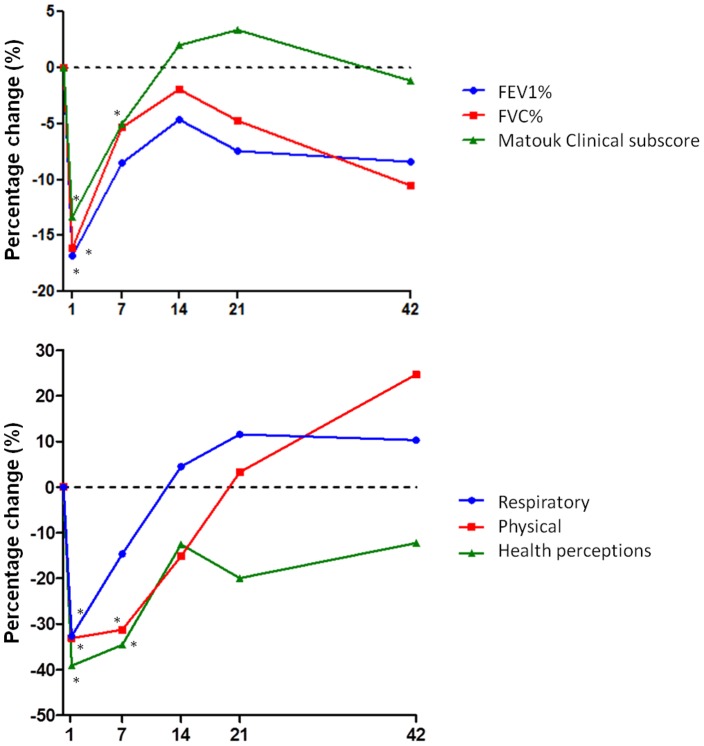
Percentage change of clinical and QOL parameters throughout PEs in CF. A) FEV1% (*blue, circles*) and FVC% (*red, squares*) were significantly reduced from baseline values at Day 1 of PE (FEV1% p = 0.001; FVC% p = 0.010). Both parameters subsequently improved with treatment however, FVC% significantly declined at Day 42 (p = 0.028). The Clinical subscore of the Matouk Disease score (*green, triangles*) was significantly decreased from baseline values on Day 1 (p = 0.000) and Day 7 (p = 0.045). See Table S1 for the results of other Matouk Disease subscores. B) QOL items also decreased with PE onset, Day 1, such as Respiratory (*blue, circles*, p = 0.002), Physical (*red, squares*, p = 0.025) and Health Perceptions domains (*green, triangles*, p = 0.000). The Physical and Health Perceptions domains remained decreased at Day 7 (p = 0.025 and p = 0.001, respectively). Other QOL domains which decreased at Day 1 include: Vitality, Health Perceptions, Social and Role (Table S1). The dotted horizontal line indicates a 0% change or no change from baseline values. * indicates a significant difference from baseline. Day 1, n = 13; Day 7, n = 12, Day 14, n = 11; Day 21, n = 9; Day 42, n = 8. Full table of results can be found in Table S1.

**Figure 3:**
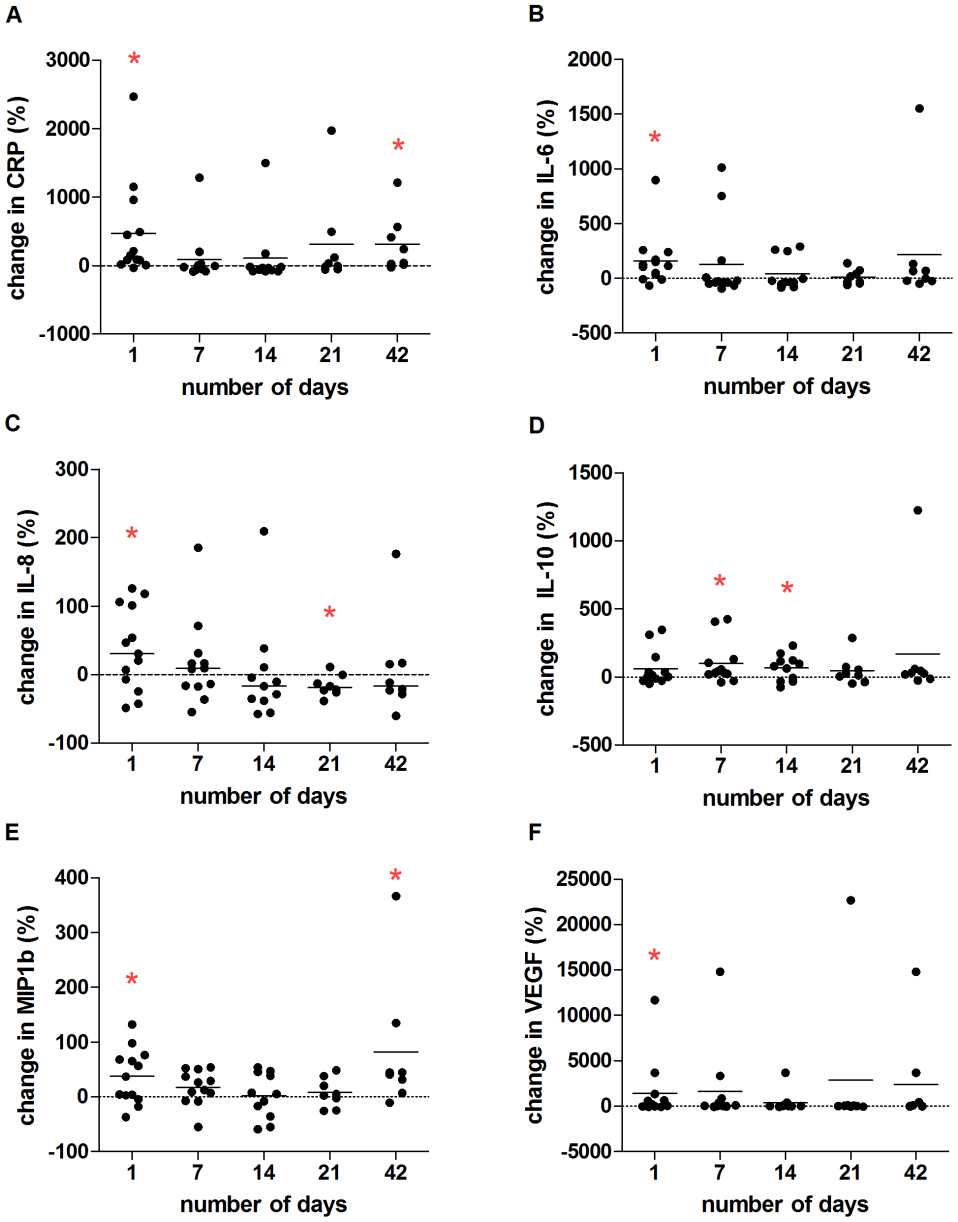
Percentage change in inflammatory markers throughout PEs in CF. Inflammatory markers were measured in blood plasma from CF patients throughout PE. The percentage change was calculated at each time point and compared to a 0% change which indicates no change from baseline (represented by dotted horizontal line). A) CRP levels were significantly increased from baseline values on Day 1 of PE (p = 0.001) and Day 42 (p = 0.039). B) IL-6 levels were significantly higher on Day 1 of PE (p = 0.006). C) IL-8 concentrations were significantly higher on Day 1 (p = 0.047) and significantly lower than baseline values after treatment on Day 21 (p = 0.022). D) IL-10 levels rose significantly on Days 7 (p = 0.021) and Day 14 (p = 0.046). E) MIP-1β increased from baseline on Days 1 (p = 0.020) and Day 42 (p = 0.023). F) VEGF levels were significantly higher on Day 1 of PE (p = 0.043). Solid horizontal lines are set at the mean * indicates a significant difference from 0% change from baseline. Day 1, n = 13; Day 7, n = 12, Day 14, n = 11; Day 21, n = 9; Day 42, n = 8. Full data set is found in Table S2.

The Cox proportional hazards and Kaplan-Meier models were used for survival analysis with time to first PE used as the outcome. For the Cox proportional hazards models, models were adjusted for age and sex. For the Kaplan-Meier method, continuous covariates were dichotomized at the median, and a log rank test was conducted (Figure 1). For comparisons of two groups, the Student's t-test or the Mann-Whitney test and Chi-square were used. For changes from baseline during PE, statistical analysis was performed using the percentage change with the one sample t-test or Wilcoxon signed-rank test (Figures 2 and 3, Table S1). The percentage change was calculated as: (Value _PE time point_−Value _baseline_)/Value _baseline_×100.

**Figure 4:**
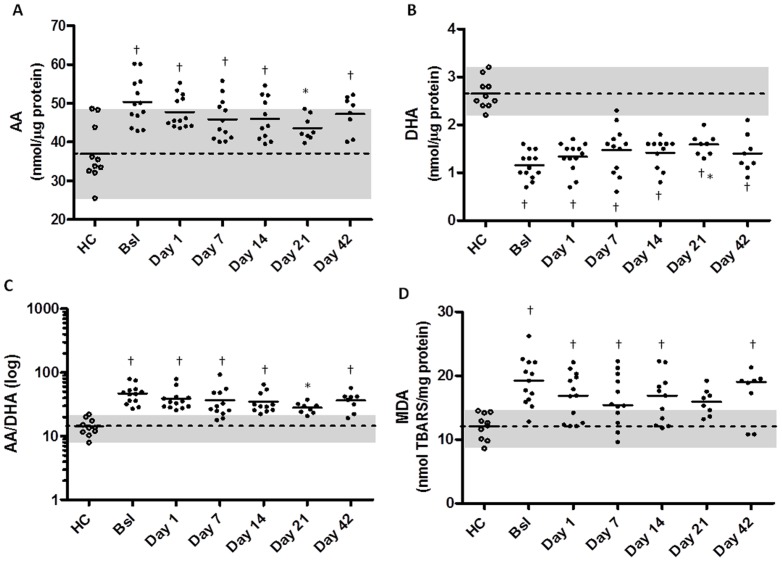
The levels of PUFA and peroxidation improve with treatment for PE in CF. PUFA concentrations were measured in blood plasma of healthy controls (HC, *white circles*) and CF patients (*black circles*) at stable disease (Bsl), at PE onset (Day 1), throughout PE treatment (Days 7, 14 and 21) and post treatment (Day 42). A) AA levels decreased during PE treatment and were significantly different from Bsl values at Day 21 (p<0.05). All PE time points including Bsl were significantly different from HC, except on Day 21 where there was no longer a difference with HC. B) DHA levels improved with PE treatment and were significantly increased from Bsl values at Day 21 (p<0.05). All PE time points including Bsl were significantly different from HC. C) Overall, the AA/DHA ratio was significantly decreased from Bsl values on Day 21 (p<0.05). All PE time points including Bsl were significantly different from HC, except on Day 21 where there was no difference with HC. D) MDA levels improved with treatment however no statistical difference was detected from Bsl. All PE time points including Bsl were significantly different from HC, except on Day 21 where there was no difference with HC. Solid lines indicate the means of the groups. Dotted lines indicate the HC mean and grey shadowing illustrates the min-max range for HC values. * represents significant difference from Bsl group using the Bonferroni post-test after ANOVA. † indicates significant difference from HC group using the Bonferroni post-test after ANOVA. Significance set as p<0.05. HC n = 10, CF n = 8–13.

The ANOVA test with Bonferroni post-tests were used to evaluate differences between the PUFA levels at all PE time points (Figure 4) and Pearson correlations were estimated for this data. Significance was set at p<0.05. See File S1 for additional description.

## Results

### Patient demographics, baseline clinical and biomarker characteristics

We prospectively followed our adult CF cohort for a total of 24 months, and 37 out of 52 patients experienced at least one PE during the follow-up period. The patient demographics and baseline values of the cohort are described in Tables 1 and 2. At baseline, CF patients that experienced at least one PE (“PE group”) had lower lung function, weight, BMI and Matouk Disease Score compared to the patients who did not have a PE (“no PE group”) (Table 1). The baseline levels of white blood cells and blood neutrophils, although still within the normal range, were higher in the “PE group”, suggesting higher levels of systemic inflammation even during a stable disease state. These patients also had lower QOL assessments for total score, Weight, Physical, Emotion, Health Perceptions, Body Image and Role items (Table 1). IL-6 and IL-10 levels were higher in the “PE group” compared to the “no PE group” (Table 2). Consistent with previously reported data from our group and other investigators[24]–[26] there was a lipid imbalance in PUFA with high AA levels and low DHA levels CF patients compared to HC. Overall the AA/DHA ratio was higher in CF patients than in HC. However, there were no differences in fatty acids between the “no PE group” and “PE group” during stable disease. Overall, these characteristics suggest that the “PE group” has more severe and active disease, and a lower QOL at baseline, compared the “no PE group”.

**Table 1:** Patient demographics and baseline values of studied parameters.

		Total CF patient group	Non-exacerbating (no PE)	Exacerbating (PE)	p-value no PE vs. PE
**Demographics**	**Number of patients**	52	15	37	
	**Sex (F/M)**	24/28	5/10	19/18	0.358
	**Age**	32.8 (1.8)	36.1 (3.4)	31.5 (2.3)	0.112
	**Age range**	18–64	18–59	18–64	
	**Weight (kg)**	60.8 (2.1)	**64.9 (2.8)**	**59.1 (2.6)**	0.031*
	**BMI (kg/m^2^)** (NR: 18.5–24.9)	21.9 (0.6)	**23.4 (0.7)**	**21.3 (0.7)**	0.009*
**Clinical assessments**	**FEV1% predicted (%)**	63.0 (3.8)	**78.5 (5.0)**	**56.8 (4.6)**	0.009*
	**FVC% predicted (%)**	79.8 (3.7)	**95.1 (5.2)**	**73.6 (4.4)**	0.008*
	**White blood cells (10^9^/L)** (NR: 4.8–10.8)	9.4 (0.4)	**8.1 (0.6)**	**10.0 (0.4)**	0.018*
	**Neutrophils (10^9^/L)** (NR: 1.6–7.7)	6.7 (0.3)	**5.6 (0.5)**	**7.1 (0.4)**	0.033*
	**Eosinophils (10^9^/L)** (NR: 0.0–0.5)	0.2 (0.02)	0.2 (0.07)	0.2 (0.01)	0.562
	**Platelets (10^9^/L)** (NR: 140–440)	284.6 (8.4)	**244.0 (14.6)**	**301.6 (9.0)**	0.001*
**Matouk Disease Score**	**Clinical** (0–50 points)	36.3 (0.6)	**39.3 (1.0)**	**35.1 (0.7)**	0.002*
	**PFT** (0–25 points)	16.6 (0.9)	**19.9 (1.1)**	**15.2 (1.0)**	0.018*
	**CXR** (0–25 points)	16.5 (0.4)	**18.2 (0.9)**	**15.8 (0.4)**	0.008*
	**Complication** (0–37 points)	2.6 (0.5)	**0.3 (0.2)**	**3.5 (0.7)**	0.000*
	**Total**	66.9 (2.0)	**77.3 (2.3)**	**62.6 (2.4)**	0.001*
**Quality of life**	**Weight**	64.1 (5.2)	**84.5 (5.5)**	**55.9 (6.5)**	0.016*
	**Respiratory**	68.3 (2.3)	74.8 (4.5)	65.6 (2.6)	0.072
	**Digestion**	78.0 (2.5)	84.5 (4.0)	75.4 (3.1)	0.064
	**Physical**	71.8 (3.4)	**83.3 (3.8)**	**67.2 (4.4)**	0.041*
	**Vitality**	64.4 (2.2)	67.8 (4.4)	63.1 (2.6)	0.341
	**Emotion**	81.7 (2.2)	**90.7 (2.6)**	**78.0 (2.7)**	0.008*
	**Eating**	90.3 (2.3)	91.1 (4.8)	89.9 (2.7)	0.537
	**Treatment burden**	66.2 (3.4)	74.8 (6.7)	62.8 (3.9)	0.113
	**Health Perceptions**	69.5 (2.8)	**80.8 (4.0)**	**54.9 (3.3)**	0.009*
	**Body Image**	73.5 (3.5)	**88.9 (3.1)**	**67.3 (4.3)**	0.004*
	**Social**	74.6 (2.5)	81.5 (4.4)	71.8 (2.9)	0.074
	**Role**	82.8 (2.8)	**91.7 (3.0)**	**79.2 (3.6)**	0.044*
	**Total** (0–1200 points)	885.2 (21.8)	**994.2 (28.9)**	**841.0 (25.1)**	0.001*

Data represented as mean (SEM). * p-value designates significant statistical difference between non-exacerbating (no PE) and exacerbating patients (PE) using Student's t-test or Mann-Whitney t-test when values were not normally distributed. NR indicates normal range for cell counts. BMI normal range from Health Canada (http://www.hc-sc.gc.ca).

**Table 2:** Baseline values of inflammatory markers and fatty acids.

	Healthy controls (HC) n = 3–11	Total CF patients group (CF) n = 52	Non-exacerbating CF patients (no PE) n = 15	Exacerbating CF patients (PE) n = 37
**CRP** (mg/L)	2.0 (1.1)	7.4 (1.0)	6.04 (1.7)	8.0 (1.2)
**IL-1β** (pg/ml)	3.8 (2.7)	0.9 (0.1)	0.79 (0.2)	0.9 (0.1)
**IL-6** (pg/ml)	2.7 (1.1)	5.2 (1.2)	2.93 (0.6)	**6.0 (1.6)†**
**IL-8** (pg/ml)	3.6 (0.9)	5.0 (0.5)	4.08 (0.6)	5.8 (0.6)
**IL-10** (pg/ml)	2.1 (0.9)	29.1 (3.2)*	19.04 (2.6)	**38.8 (7.2)†**
**MIP-1β** (pg/ml)	31.8 (6.9)	24.3 (1.6)	24.83 (3.2)	24.2 (1.9)
**TNF** (pg/ml)	5.3 (0.5)	3.8 (0.3)*	3.45 (0.5)	3.9 (0.4)
**VEGF** (pg/ml)	129.5 (46.7)	95.7 (16.5)	82.45 (17.3)	101.0 (22.2)
**AA** (nmol/µg of protein)	37.0 (2.4)	49.5 (0.8)*	48.45 (1.5)	49.9 (1.0)
**DHA** (nmol/mg of protein)	2.7 (0.1)	1.2 (0.1)*	1.30 (0.1)	1.2 (0.1)
**AA/DHA ratio**	14.4 (1.4)	48.8 (4.6)*	40.92 (3.8)	51.8 (6.1)

Data represented as mean (SEM). * p-value represents statistically significant difference between HC and CF groups. † represents statistically significant difference between “no PE” and “PE” groups. Significance was set at p<0.05.

### Worse clinical disease severity and activity, and QOL are associated with PE events

Using the Cox proportional hazards model adjusting for age and sex, we tested the association between our markers and the risk of PE (Table 3). Better lung function (FEV_1_% and FVC%) and higher Clinical, PFT, CXR subscores and total Matouk Disease Score were associated with a lower risk of PE. A lower Complications subscore was associated with lower risk of PE. The Matouk Disease Score incorporates patients' symptoms as well as physician-recorded clinical parameters and other complications adding to disease activity beyond spirometric evaluations. Thus a high degree of disease severity as measured by lung spirometry was associated with high risk of PE as was high disease activity assessed by the Matouk Disease Score. The analysis also showed that low CFQ-R symptom scores (Weight and Respiratory) and low QOL domains (Physical, Vitality, Health Perceptions and Role) indicated higher risk of PE. No inflammatory markers or PUFA were found to be associated with risk of PE using the Cox proportional hazard model.

**Table 3:** Markers influencing time to first PE during stable disease.

Marker		Hazard ratio^1^	95% hazard ratio confidence limits	p-value
**Clinical assessment**	FEV1% predicted	0.97	0.94–0.99	0.006
	FVC% predicted	0.97	0.95–0.99	0.010
**Matouk Disease Score**	Clinical	0.74	0.63–0.86	0.000
	PFT	0.88	0.80–0.98	0.015
	CXR	0.71	0.50–0.94	0.017
	Complication	1.33	1.17–1.51	0.000
	Total	0.92	0.88–0.96	0.000
**Quality of life**	Weight	0.97	0.95–0.99	0.006
	Respiratory	0.96	0.92–0.99	0.024
	Physical	0.96	0.93–0.99	0.003
	Vitality	0.96	0.93–1.00	0.043
	Health perceptions	0.95	0.92–0.99	0.005
	Role	0.96	0.94–0.99	0.020
	Total	0.99	0.99–1.00	0.004

1 HR<1 is associated with lower risk of future PE.

To illustrate these relationships, we used the Kaplan-Meier survival model, with patients dichotomized based on median values (Figure 1). We obtained similar results to the Cox proportional hazards model. Lower lung function (FEV_1_%, Figure 1A and FVC%, Figure 1B), worse subscores of the Matouk Disease Score (low Clinical subscore, Figure 1C and high Complications subscore Figure 1D, data not shown for other subscores) and low scores for the CFQ-R QOL domains Physical and Health Perceptions were also related to a higher risk of a future PE (Figures 1E and 1F, respectively). No baseline inflammatory markers or PUFA were associated with increased risk of PE events, although there was a trend with higher CRP levels (median 5.3 mg/L, p = 0.0537, data not illustrated).

### Changes in clinical parameters and QOL during PE

We performed a second study with 13 patients to evaluate whether any of our markers at the end of PE treatment would be predictive of an early re-exacerbation. We first calculated the percentage change at each time point for each patient compared to their baseline values obtained during a period of stable disease (Figure 2 and full data set is presented in Table S1). As expected, lung function was most reduced at Day 1 of a PE (−16.8% FEV_1_% and −16.1% FVC% compared to baseline), and steadily improved over the course of PE treatment lasting either 14 or 21 days (Figure 2A). FEV_1_% and FVC% approached pre-PE baseline values at Day 14. However, both values decreased again by Day 21 and 42 where FVC% values were 10.5% lower than baseline values (p = 0.028)(Figure 2A). At the onset of PE (Day 1), all components of the Matouk Disease Score worsened compared to baseline (Clinical subscore illustrated in Figure 2A). The Clinical subscore steadily improved with PE treatment where it was 3.4% above baseline on Day 21. The CXR subscore did not decrease at PE onset, however showed a trend toward improvement on Day 21, attesting to the limited sensitivity of the Bradsfield radiologic scoring to capture small CXR changes (Table S1). The QOL evaluation revealed that patients recognized a decline of their health on Day 1 with improvements on Days 7, 14, 21 and 42 (Respiratory, Physical and Health Perceptions illustrated in Figure 2B).

### Changes in inflammatory markers during PE

In previous studies, inflammatory markers such as CRP, IL-1β, IL-6, IL-8 and VEGF were found to increase with PE onset and to respond to antibiotic treatment for PEs [14], [15], [27]–[32]. We calculated the percentage change for the concentrations of inflammatory markers at each time point, in comparison to baseline values for each patient. In general, we also found PE onset induced an inflammatory response which returned to baseline values toward the end of treatment (Days 14 and 21). Once off aggressive treatment for PE, inflammatory markers tended to increase again in some patients at Day 42. More specifically, we found the elevated CRP levels from baseline values on Day 1 decreased during treatment and significantly worsened once again post-treatment by Day 42 (Figure 3A). Similarly, IL-6, IL-8, MIP-1β and VEGF also increased on Day 1 and improved over the course of treatment (Figure 3B, C, E, F, respectively). IL-10 increased on Days 7 and 14 from baseline values, suggesting that anti-inflammatory mechanisms were activated (Figure 3D). Interestingly, MIP-1β levels significantly increased again post-treatment on Day 42 (Figure 3F). All other inflammatory markers tended to increase at Day 42, although with a large variation between patients. IL-1β and TNF were not significantly changed throughout PE (Table S2).

### Improvements in PUFA levels and lipid peroxidation during PE

A hallmark of CF disease is the imbalance in PUFAs with high levels of AA and low levels of DHA[24]-[26], [33], [34]. AA is pro-inflammatory and its metabolites include prostaglandins and eicosanoids which are also increased in CF[35], [36]. DHA is anti-inflammatory and its metabolites include resolvins and protectins. This imbalance in PUFAs may contribute to the inflammatory status observed in CF[37], [38]. In fact, our previous studies in a CF mouse model showed improvements in AA and DHA after treatment with fenretinide, a semi-synthetic retinoid, which were concurrent with reductions in inflammatory markers and better clearance of lung infections[25], [39].

Unexpectedly, we found improvements in the PUFA imbalance compared to baseline on Day 21 for both AA (Figure 4A) and DHA (Figure 4B). At this time point, AA levels dropped to normal values, the AA/DHA ratio improved (Figure 4C), and both were no longer significantly different from HC concentrations. It is important to note that these improvements were not permanent as AA levels and AA/DHA ratios increased by Day 42. Lipid peroxidation decreased with treatment for PE and, MDA levels were no longer different from HC at Day 21(Figure 4D). We found a significant positive correlation between MDA and AA in plasma at the end of treatment (r = 0.6917, p = 0.013) and an inverse correlation between MDA and DHA, however this trend did not reach significance (r = −0.3392, p = 0.290).

### Potential promising markers of early re-exacerbation

To determine whether any of our markers assessed on the last day of treatment (either Day 14, n = 4, or Day 21, n = 8) could indicate an early re-exacerbation, the patient cohort was divided based on whether their next PE was under or over 42.5 days from the last day of treatment, which was the median number of days for the group (Table 4). “Early PE” refers to the patient group which had their next PE less than 42.5 days after the last day of treatment, while the group which developed no subsequent PE or a second PE more than 42.5 days after the last day of treatment was called “Late PE”. One patient was excluded from the analysis since information on their next PE was not available, thus 12 patients were included. The “Early PE” group had a trend towards lower lung spirometry values, total Matouk Disease Score and QOL total score at end of treatment compared to the “Late PE” group, but this did not result in a statistical significance. More importantly, we found increased levels of inflammatory markers in “Early PE” patients with significantly higher CRP and IL-8, indicating that these markers may contribute to assessing which patients could rapidly re-exacerbate.

**Table 4:** Markers associated with early re-exacerbation at the end of PE treatment.

	Early PE (n = 6)	Late PE (n = 6)	p- value
Days until next PE mean (min – max)	17.3 (1–29)	142.5 (56–365)	N/A
FEV1% predicted (%)	38.5 (7.2)	50.0 (8.5)	0.328
FVC% predicted (%)	56.5 (9.9)	64.0 (10.0)	0.606
Matouk Total score	46.5 (6.2)	56.8 (3.7)	0.180
QOL Total score	752.0 (86.7)	765.5 (62.8)	0.902
CRP (mg/L)	42.5 (26.1)	5.2 (2.7)	0.045*
IL-6 (pg/ml)	4.9 (1.6)	2.6 (0.6)	0.199
IL-8 (pg/ml)	3.9 (0.4)	2.7 (0.3)	0.034*
IL-10 (pg/ml)	24.4 (2.5)	77.0 (37.8)	0.195

Data presented as mean (SEM) unless specified. * indicates significant difference between the groups using Student's t-test or Mann-Whitney test if values were not normally distributed.

## Discussion

The importance of PEs in CF disease progression is well established however the triggers of these events are still being understood which limits the means for prevention. Retrospective studies have identified patient groups at risk of PEs such as those with liver disease, CF related diabetes, low FEV_1_ values [5], [12]. However, the difficulty remains in determining on an individual basis which patient has a high risk of a future PE.

Our prospective study is unique for two main reasons: 1) the large panel of data that were collected at stable disease and 2) the extensive sampling throughout PEs. With these results, we assessed markers predicting future PE in two distinct cases: from stable disease and at the last day of treatment for PE.

### Assessing risk from stable disease

Patients who developed PEs had worse baseline disease severity (based on lung spirometry and BMI), greater baseline disease activity (based on the Matouk Disease Score) and worse self-reported QOL at stable disease. Interestingly, this was associated with higher inflammatory markers (Tables 1 and 2). Using survival models, the clinical (lung spirometry and Matouk Disease score) and QOL assessments were predictive of future PE with better scores associated with lower risk of PE (Figure 1 and Table 3). The inflammatory markers we assessed were not associated with future PE when measured at stable disease. Noteworthy, there was a trend with low CRP associated with low risk of PE, which merits further investigation with a larger cohort of patients. Monitoring changes in these markers may lead to an early recognition of PEs which would allow for earlier intervention. This, in turn, would reduce the impact of heightened and prolonged inflammation on lung tissue.

Presently, there are no known methods which could prevent the occurrence of PEs. Current treatments for CF have shown impacts on reducing the number of PEs, such as routine inhaled antibiotic therapy for infected patients, human recombinant deoxyribonuclease and inhaled hypertonic saline [40]. Some CF centers advocate the routine use of IV antibiotics for two weeks every three months, rather than in only response to PE symptoms. They report improved survival rates using this protocol, however its impact on the rate of PEs is unclear [41], [42]. Elborn et al. reported no significant effect of elective IV antibiotics every three months (four/year) in a randomized control trial compared to treating patients because of PEs. Patients in the control group were prescribed IV antibiotics three times per year while 40% of the elective treatments were in response to PEs. Thus it is unclear whether elective IV antibiotics had a significant impact on reducing the rate of PE [43], [44]. However, the number of PEs in each group were not directly compared, thus based on the reports currently available, it is difficult to conclude whether the rate of PEs can be reduced by preventative aggressive antibiotics. These treatments were assigned at specific times (every three months) which may come too early to prevent PEs. Rather patients may benefit from obtaining IV antibiotics as soon as a change is detected in their clinical status or QOL assessments. Previous studies have shown that CF patients who recorded their lung spirometry using a daily diary had lower rates of lung function decline than those who did not [45]. A new study by Lechtzin et al. looking at twice weekly electronic symptom recording by CF patients may help determine the usefulness of a more frequent symptom and lung function monitoring for early recognition of PEs[7].

### Prediction from end of treatment

Each PE significantly decreases lung function in CF patients, even more with PEs in rapid succession [4]. In our study, we found that higher inflammation at the end of treatment may be a better indication of early re-exacerbation rather than clinical or patient QOL assessments (Table 4). The few prospective studies looking at predictors of future PEs used measurements at the end of PE treatment. CRP was previously shown to be correlated to the number of days until the next PE [14], a finding not universally reported[15]. Regardless of the discrepancies, these studies showed the usefulness of inflammatory markers such as CRP and calprotectin in assessing risk of future PE [14], [15]. Sequeiros et al. also showed that early re-exacerbation was correlated with more symptoms after 14 days of treatment such as cough, sputum production, breathlessness and fatigue [14].

We found that FEV_1_% values tended to be lower in patients that quickly re-exacerbated, and due to our small patient group, we cannot disregard this as a marker of recurring PE. Thus, inflammatory markers such as CRP and IL-8, in conjunction with patient reported symptoms, clinical evaluations and spirometry, could be additional indicators of a recurring PE. And, although symptoms and clinical picture (lung function and Matouk Disease score) have returned to baseline at the end of treatment, patients may still be experiencing some level of unresolved inflammation indicating the PE has not been cleared completely. There has been evidence that extending the course of antibiotics may only offer a small improvement in symptoms but not lung function or inflammation[46]. In these cases, perhaps a change in antibiotics and/or the use of anti-inflammatory agents to aid in the resolution of inflammation would benefit the patient and prevent early re-exacerbation. However, based on our study, we cannot determine whether the unresolved inflammation itself triggers a new PE or whether it is a response to other underlying active processes such as a poorly controlled infection. We did not look at causes of PEs such as viral infections which have been reported to impact recovery from PE[9].

### Kinetics of inflammation and PUFA throughout PE

We found in general inflammatory markers increased at PE onset such as CRP, IL-6, IL-8, MIP-1β and VEGF compared to values at stable disease periods, and responded to treatment as soon as Day 7 (Figure 3). This corroborates previous findings regarding resolution of inflammation with antibiotic treatment[14], [27], [32], [47]. MIP-1β, a chemoattractant for monocytes, has rarely been assessed in CF and, to our knowledge, this is the first time reported to respond to treatment in the context of PEs in CF.

Our study design allowed us to monitor whether improvements during PE treatment are maintained after treatment was completed. Five patients out of 13 were not available for Day 42 assessments due to a re-exacerbation (n = 4) or other complications (n = 1, same patient that was excluded from the second study on markers associated with early re-exacerbation). Even with this small number of patients, we were able to observe decreases in FVC% values and increases in CRP and MIP-1β at Day 42 compared to baseline values. Due to the small sample size and large variation at this time point, we could not conclude whether these changes are indicative of future exacerbations and further studies are necessary. However, it is important to recognize that improvements at the end of treatment, a time point assessed in many PE studies, may not be representative of the patient's disease status on the long term.

Few studies have assessed the changes in PUFAs at PE onset and throughout treatment[48], [49]. Similar to our results regarding lipid peroxidation, McGrath and colleagues demonstrated a decrease MDA in CF patients after antibiotic treatment for PE[50]. The improvements in PUFA during PE occur during treatment but worsen at Day 42, when patients are no longer treated for PE. Thus the improvements in the AA/DHA ratio may be due to the effects of the treatment itself in reducing lipid peroxidation, also observed in this study. Antibiotics have been shown to protect lung epithelial cells from oxidative damage[51]. Tobramycin in particular was found to act as a potent reactive oxygen species scavenger[52]. In general, PUFAs are very susceptible to peroxidation due to their high content of double bonds compared to other types of fatty acids causing DHA to be more affected than AA[53]. The increase in anti-inflammatory DHA may contribute to the resolution of inflammation. Additionally, the reduction in lipid peroxidation may itself be a factor. Oxidized fatty acids such as DHA were found to act on Toll-Like Receptor 4 much like its ligand lipopolysaccharide activating downstream NF-κB signalling[54]. It is important to note that changes in PUFA were not permanent and routine treatments for disease maintenance do not normalize PUFA levels since CF patients at stable disease still have defects in AA and DHA levels (Figure 4 and Table 2). Currently, no antibiotic or steroid therapy for CF has proven to be successful in correcting PUFA abnormalities, which could improve disease status of patients.

### Limitations

Our study has several limitations that are important to consider. Among the 52 patients included in our analysis, we had longitudinal clinical and molecular marker measurements in only 13 patients during their PEs. Due to the extensive testing and the availability of the clinical coordinator, this was the volume of patients we could handle for the study period. However, due to the large panel of markers we assessed and the prospective nature of the study design, this study remains an important exploratory analysis generating many hypotheses about predictive markers of PE.

The median time to next PE was somewhat low suggesting that our patient group had high disease activity. Parkins et al. described that 13% of their patient cohort re-exacerbated in 45 days. They used this time point as a definition of non-response to treatment which may be another way to describe the six patients with early re-exacerbations since they had higher levels of inflammation[1]. Our Day 42 time point was also affected when it overlapped with new PEs. Noteworthy, our definition of PE was more inclusive than the other definitions used[5], [14], [27] as it included any event needing additional IV or oral therapy thereby including mild and severe types of exacerbations.

## Conclusions

This study demonstrates that monitoring changes in clinical and patient reported assessments during stable disease may help in determining which patients are at risk for PEs. Our longitudinal analysis of inflammation throughout PEs suggests that, at the end of antibiotic treatment for PE, inflammatory markers could contribute to monitoring patients at risk of early recurring PE. The imbalance in PUFA levels improve after treatment for PE possibly due to a decrease in lipid peroxidation. Regarding early re-exacerbation, our analysis reveals that CRP and IL-8 in particular can be important in assessing patients at risk of early recurring PEs, which needs to be confirmed in a larger study. The data presented here offer more insight into potential markers of PEs which, in conjunction with clinical data, may improve earlier recognition of PEs in CF.

## Supporting Information

File S1 **Additional explanation of methods including a full description of the scoring method and points allocated for the Matouk Disease Score, and additional information regarding the statistical methods used.**
(DOCX)

Table S1 **Percentage change of clinical parameters throughout exacerbations in CF.** Includes percentage change, standard errors and statistical evaluation of clinical parameters, Matouk Disease Score and QOL assessments throughout PE time points.
(DOCX)

Table S2 **Percentage change of inflammatory markers during PE.** Includes percentage change, standard errors and statistical evaluation of inflammatory markers throughout PE time points.
(DOCX)
