# Supplementary material for: Candidate Markers Associated with the Probability of Future Pulmonary Exacerbations in Cystic Fibrosis Patients
Source: PLoS One. 2014 Feb 12;9(2):e88567. doi: 10.1371/journal.pone.0088567 (PMC3922941; doi:10.1371/journal.pone.0088567)
Supplement: File S1 — Additional explanation of methods including a full description of the scoring method and points allocated for the Matouk Disease Score, and additional information regarding the statistical methods used. (DOCX) [file pone.0088567.s001.docx]

**Matouk Disease Score**

Description of the Matouk Disease Score extracted from “Internal consistency reliability and predictive validity of a modified N. Huang clinical scoring system in adult cystic fibrosis patients.” Matouk E, Ghezzo RH, Gruber J, Hidvegi R, Gray-Donald K. *Eur Respir J*. 1997 Sep;10(9):2004-13.

***Clinical subscore*** *(50 points total – higher score reflects healthier patient)*

1. Weight

5 = normal weight (ideal body weight)

4 = 85–90% ideal body weight

3 = 80–84% ideal body weight

2 = 75–79% ideal body weight

1 = <75% ideal body weight

2. Weight change (compared to the best previous over the past 12 months)

5 = no weight loss

4 = weight loss 2–3%

3 = weight loss 4–5%

2 = weight loss 6–9%

1 = weight loss ≥10%

3. Dyspnoea

5 = dyspnoea 0: not troubled with breathlessness except with strenuous exercise

4 = dyspnoea 1: troubled with shortness of breath when hurrying on the level or walking up a slight hill

3 = dyspnoea 2: walks slower than people of the same age on the level because of shortness of breath or has to stop or breathing when walking at own pace on the level

2 = dyspnoea 3: stops for breath after walking about 100 m or after few minutes on the level

1 = dyspnoea 4: too breathless to leave the house or breathless when dressing or undressing

4. Cough

5 = no cough even with physical therapy and postural drainage

4 = cough with physical therapy and postural drainage or dry occasional cough

3 = infrequent productive cough 1–5 times/day

2 = frequent productive cough more than 5 times/day including night cough

1 = frequent severe productive cough or coughing spells once each 30–60 min

5. Sputum

5 = none most of the time

4 = clear or white

3 = yellow or beige

2 = pale green

1 = dark green or brown or dark grey

6. Physical examination (air entry/ crackles/ hyperinflation)

5 = normal air entry, or lungs clear, or no hyperinflation

4 = mildly reduced air entry, or lungs clear, or mild hyperinflation

3 = moderately reduced air entry, or localized inspiratory crackles and/or one area of bronchial breathing or moderate hyperinflation

2 = moderately severe reduction in air entry, or 2–3 areas of inspiratory crackles, and/or areas of bronchial breathing, or moderately severe hyperinflation

1 = severe reduction in air entry, or diffuse inspiratory crackles and/or more than areas of bronchial breathing or severe hyperinflation

The score chosen will correspond to only one or another item on the same line.

7. Respiratory rate/breathing pattern/cardiac frequency (respiratory rate combined score)

5 = normal respiratory frequency for age, or normal breathing pattern, or normal cardiac frequency

4 = slightly faster than normal respiratory frequency (12–14 breaths·min-1) or no use of accessory muscles and or no intercostal retraction or normal cardiac frequency

3 = moderate increase (14–20 breaths·min-1), or mild use accessory muscles and or mild intercostal retraction, or mild increase cardiac frequency 90–100 beats·min-1.

2 = moderately severe tachypnoea (>20–26 breaths·min-1), or moderate use of accessory muscles and/or moderate intercostal retraction, or moderate increase in cardiac frequency to 100–120 beats·min-1.

1 = marked tachypnoea (>26 breaths·min-1), or severe use of accessory muscles and/or severe intercostal retraction

and/or interference with carrying out regular conversation and/or paradoxical abdominal movements, or severe increase cardiac frequency >120 beats·min-1. The score chosen will correspond to only one or another

item on the same line.

8. Culture

5 = Normal throat flora

4 = *Staphylococcus aureus* and/or other gram-positive cocci with or without *Haemophilus influenzae*

3 = *Pseudomonas aeruginosa*, nonmucoid or *Pseudomonas fluorescens* or nonmucoid Pseudomonas spp.

2 = *Pseudomonas aeruginosa*, mucoid and or mucoid *Pseudomonas* spp., *Stenotrophomonas maltophilia*, *Serratia*

*marscecens*, *Klebsiella ozaenae*, *Escherichia coli*, *Acinetobacter* spp. and *Alcaligenes* spp.

1 = *Burkholderia cepacia* and/or the presence of multiply resistant *Pseudomonas aeruginosa* or species nonmucoid or mucoid

In the presence of multiple organisms, the organism in the worst category will dictate the score.

9. Appetite

5 = greater than normal (two servings of food at each meal)

4 = good appetite (consuming slightly more than a regular serving of food at each meal)

3 = average appetite (consuming a regular serving of food at each meal)

2 = less than average appetite (consuming slightly less than a regular serving of food at each meal)

1 = poor appetite

10. General condition (This score representes the clinician's assessment of the patient's general condition)

5 = cheerful, energetic

4 = pleasant, sociable with peers, but appearing chronically ill

3 = depressed, irritable, uncooperative, uncomfortable appearance

2 = very sick and depressed

1 = bedridden

***Pulmonary function subscore*** *(25 points – higher score reflects healthier patient)*

5 4 3 2 1

FVC (% pred) ≥80% 65–80% 50–64% 35–49% <35%

FEV1 (% pred) ≥80% 60–80% 40–59% 20–39% <20%

FEV1/FVC ≥75% 65–75% 55–64% 45–54% <45%

FEF25–75 (% pred) ≥60% 45–60% 30–44% 20–29% <20%

FEF50 (% pred) ≥60% 45–60% 30–44% 20–29% <20%

RV/TLC <25% 25–29% 30–39% 40–49% ≥50%

***Radiographic subscore*** *(25 points – higher score reflects healthier patient)*

1. Air trapping

5 = none

4 = minimal hyperinflation

3 = mild diffuse hyperinflation with increase in anteroposterior (A-P) diameter and low diaphragm

2 = moderately diffuse hyperinflation plus change in A-P diameter and low diaphragm

1 = severe hyperinflation with flat diaphragms and dark square chest, sternal bowing with or without thoracic kyphosis

2. Linear markings (single or parallel line densities (tram lines), or end on circular densities due to bronchial wall

thickening (ring shadows))

5 = none

4 = minimal impression of increased perihilar markings

3 = definite thickening confined to central and two or less peripheral areas

2 = diffuse markings extending to the periphery of the lung

1 = severe, diffuse markings ("mostly white streaks")

3. Nodular cystic lesions (discrete, small, rounded densities about 0.5 cm in diameter or larger, radiolucent or radio-opaque).

5 = none

4 = few lesions

3 = multiple lesions, but localized

2 = multiple lesions diffusely scattered

1 = almost honeycomb appearance

4. Parenchymal lesions (segmental or lobar atelectasis or consolidation including pneumonia)

5 = none

4 = single, small area involved

3 = consolidation of one or more segments of a lobe

2 = consolidation including two different lobes

1 = consolidation involving more than two lobes

5. General impression (judgement of overall severity of radiographic changes)

5 = normal appearing

4 = slightly abnormal involving one area or one quadrant

3 = mildly, but diffusely, abnormal

2 = moderately severe change

1 = severe changes present (including pneumothorax,

congestive heart failure)

***Complications subscore*** *(maximum = 37 points – lower score reflects healthier patient).*

Subtract points from total score for each serious complication.

1. Pulmonary exacerbation requiring therapy (max 7)

a) Present or past 3 months 5 points

b) Past year 2 points

2. Pneumothorax (max 5)

a) Past 6 months or recurrent 5 points

(*i.e*. ≥2 episodes over any period of time)

b) Ever 2 points

3. Haemoptysis (max 5)

a) Massive past 6 months 5 points

more than 6 months ago 2 points

b) Minor in the past 3 months 1 point

4. Respiratory failure (max 10)

*P*a,O2 < 60 mmHg; ≥ 55 mmHg 4 points

*P*a,O2 < 55 mmHg; ≥ 50 mmHg 6 points

*P*a,O2 <50 mmHg 8 points

Any of above with hypercapnia 9 points

Any above + previous intubation (in past year) 10 points

5. Cardiac enlargement or congestive heart failure (max 5)

cardiac enlargement 3 points

congestive heart failure 5 points

6. Pulmonary surgery (max 5)

≤ one lobe 3 points

> one lobe 5 points

***Total score*** = [clinical subscore (max 50 points) + radiographic subscore (max 25 points) + pulmonary function subscore (max 25 points)] - complications subscore (max 37 points)

**Statistical analysis.**

The SAS program (Toronto, On, Canada) was used to assess the relationship between the markers at baseline and PE events. First, we used the Cox proportional hazards model adjusted for age and sex to determine the relationships of our markers to the time to first PE. The Kaplan-Meier survival model with log rank test was used to illustrate the survival analysis data with continuous covariates were dichotomized at the median. All other statistical analyses were performed with Prism 5 (GraphPad Software, LaJolla, CA, USA). We used the D’Agostino and Pearson omnibus normality test to assess normality, the Student’s t-test or the Mann-Whitney test to compare two groups, and Chi-square test for categorical values. The percentage change was calculated from baseline values and the one sample t-test or Wilcoxon signed-rank test was used to determine changes from baseline. Positive values indicate increases and negative values indicate decreases from baseline. Fatty acid data are presented with reference to normal values and the ANOVA test was used to evaluate differences between the groups. Correlations were performed using the Pearson test. Significance was set at p < 0.05.
