# Supplementary material for: Candidate Markers Associated with the Probability of Future Pulmonary Exacerbations in Cystic Fibrosis Patients
Source: PLoS One. 2014 Feb 12;9(2):e88567. doi: 10.1371/journal.pone.0088567 (PMC3922941; doi:10.1371/journal.pone.0088567)
Supplement: Table S1 — Percentage change of clinical parameters throughout exacerbations in CF. Includes percentage change, standard errors and statistical evaluation of clinical parameters, Matouk Disease Score and QOL assessments throughout PE time points. (DOCX) [file pone.0088567.s002.docx]

**Table S1. Percentage change of clinical parameters throughout exacerbations in CF**

|  |  | **Day 1**  **n = 13** | **p-value** | **Day 7**  **n = 12** | **p-value** | **Day 14**  **n = 11** | **p-value** | **Day 21**  **n = 8** | **p-value** | **Day 42**  **n = 8** | **p-value** |
| --- | --- | --- | --- | --- | --- | --- | --- | --- | --- | --- | --- |
| **Clinical parameters** | **FEV1% predicted** | -16.8 (3.9) | **0.001*** | -8.5 (4.2) | 0.068 | -4.7 (5.3) | 0.397 | -7.4  (3.9) | 0.141 | -8.4  (5.3) | 0.159 |
|  | **FVC% predicted** | -16.1 (5.3) | **0.010*** | -5.3 (4.3) | 0.240 | -1.9 (5.2) | 0.719 | -4.7  (4.3) | 0.150 | -10.5 (3.8) | **0.028*** |
|  | **White blood cells** | 28.4 (12.4) | **0.002*** | 30.9 (11.7) | **0.023*** | 35.5  (21.4) | 0.175 | 11.4 (15.5) | 0.547 | 0.9  (8.2) | 0.920 |
|  | **Neutrophils (%)** | 45.3 (19.3) | **0.005*** | 36.1 (12.9) | **0.018*** | 43.8 (28.1) | 0.150 | 17.5  (25.0) | 0.742 | 6.3 (12.2) | 0.621 |
|  | **Eosinophils (%)** | 37.0 (16.2) | **0.041*** | 79.8 (26.2) | **0.011*** | 73.1 (26.3) | **0.020*** | 177.8 (62.0) | **0.016*** | 26.3 (24.5) | 0.483 |
|  | **Platelets (%)** | 4.5  (5.9) | 0.461 | 5.7 (5.6) | 0.328 | 4.9 (5.6) | 0.403 | -0.02 (4.6) | 0.996 | 3.1  (9.2) | 0.743 |
| **Matouk Disease Score** | **Clinical** | -13.3 (2.5) | **0.000*** | -5.0 (1.9) | **0.045*** | 2.0 (1.8) | 0.273 | 3.4  (4.2) | 0.446 | -1.1  (6.0) | 0.856 |
|  | **PFT** | -11.0 (3.5) | **0.009*** | -6.3 (4.0) | 0.150 | -7.4 (4.7) | 0.148 | -6.8  (6.7) | 0.176 | -5.6  (6.3) | 0.400 |
|  | **CXR** | -1.9  (2.0) | 0.375 | 0.2 (2.4) | 0.949 | -0.5 (2.4) | 0.853 | 3.5  (1.8) | 0.099 | -1.5  (2.9) | 0.615 |
|  | **Compli-cations** | 169.9 (67.1) | **0.006*** | 171.6 (73.0) | **0.004*** | 197.0 (76.9) | **0.009*** | 203.9 (104.2) | **0.036*** | 216.6 (103.5) | **0.022*** |
|  | **Total** | -19.0 (1.8) | **<0.000*** | -11.2 (2.3) | **0.001*** | -7.6 (1.8) | **0.002*** | -5.6  (2.1) | **0.016*** | -10.2 (4.1) | **0.042*** |
| **Quality of life** | **Weight** | -6.4  (9.4) | 0.505 | 7.0 (16.6) | 0.683 | 3.0 (17.0) | 0.862 | 6.2  (6.2) | 1.000 | 37.5 (24.6) | 0.174 |
|  | **Respiratory** | -32.7 (5.6) | **0.002*** | -14.6 (9.5) | 0.240 | 4.5 (10.0) | 0.844 | 11.6 (9.9) | 0.280 | 10.3 (9.6) | 0.438 |
|  | **Digestion** | 9.5  (7.7) | 0.362 | 3.6 (6.1) | 0.352 | 9.3 (7.0) | 0.214 | 23.9 (10.4) | 0.055 | 18.1 (11.7) | 0.166 |
|  | **Physical** | -33.1 (15.4) | **0.025*** | -31.3 (10.1) | **0.010*** | -15.2 (6.3) | 0.146 | 14.1 (21.4) | 0.531 | 24.7 (48.2) | 0.195 |
|  | **Vitality** | -34.7 (5.2) | **0.002*** | -34.9 (7.9) | **0.001*** | -18.0 (5.5) | **0.009*** | -17.9 (13.1) | 0.213 | -17.1 (12.1) | 0.200 |
|  | **Emotion** | -9.8  (8.8) | 0.288 | -4.0 (6.2) | 0.531 | 1.5 (6.5) | 0.826 | -3.5  (7.8) | 0.672 | 6.9  (7.2) | 0.469 |
|  | **Eating** | -11.7 (7.8) | 0.123 | -21.1 (5.9) | **0.004*** | -11.1 (6.4) | 0.115 | -1.6  (4.6) | 0.745 | -8.3 (10.0) | 0.434 |
|  | **Tx burden** | -3.9  (5.8) | 0.291 | -18.5 (7.1) | **0.024*** | -11.7 (5.6) | 0.064 | -26.6 (11.5) | **0.036*** | 6.9 (10.6) | 1.000 |
|  | **Health Perceptions** | -39.1 (7.3) | **0.000*** | -34.6 (7.5) | **0.001*** | -12.5 (6.4) | 0.079 | -18.3 (11.6) | 0.160 | -12.3 (12.4) | 0.357 |
|  | **Body image** | 2.7 (20.5) | 0.844 | 11.4 (13.8) | 0.624 | -3.6 (11.6) | 0.762 | 11.1 (22.1) | 0.633 | 1.3 (17.5) | 0.943 |
|  | **Social** | -14.3 (4.0) | **0.004*** | -16.7 (5.5) | **0.011*** | -14.3 (8.9) | 0.203 | -14.2 (11.7) | 0.266 | -7.4  (7.9) | 0.375 |
|  | **Role** | -11.4 (8.4) | 0.196 | -22.3 (8.9) | **0.019*** | -2.2 (15.3) | 0.641 | -9.8  (6.6) | 0.279 | -5.2 (10.5) | 0.638 |
|  | **Total** | -17.4 (4.2) | **0.001*** | -24.2 (7.8) | **0.001*** | -7.7 (3.1) | **0.035*** | -2.6  (4.6) | 0.585 | 1.0  (7.3) | 0.844 |

Data represented as means of percentage changes (SEM). * p-value indicates significant statistical difference between time point vs. baseline values as determined using one sample t-test or Wilcoxon Signed Rank test when values were not normally distributed.
