# Supplementary material for: Candidate Markers Associated with the Probability of Future Pulmonary Exacerbations in Cystic Fibrosis Patients
Source: PLoS One. 2014 Feb 12;9(2):e88567. doi: 10.1371/journal.pone.0088567 (PMC3922941; doi:10.1371/journal.pone.0088567)
Supplement: Table S2 — Percentage change of inflammatory markers during PE. Includes percentage change, standard errors and statistical evaluation of inflammatory markers throughout PE time points. (DOCX) [file pone.0088567.s003.docx]

**Table S2. Percentage change of inflammatory markers during PE**

|  | **Day 1**  **n = 13** | **p-value** | **Day 7**  **n = 12** | **p-value** | **Day 14**  **n = 11** | **p-value** | **Day 21**  **n = 8** | **p-value** | **Day 42**  **n = 8** | **p-value** |
| --- | --- | --- | --- | --- | --- | --- | --- | --- | --- | --- |
| **CRP**  **(%)** | 470.9 (196.2) | **0.001*** | 85.7 (111.5) | 0.380 | 107.6 (141.0) | 0.320 | 309.8  (245.8) | 0.461 | 310.1 (149.4) | **0.039*** |
| **IL-1β (%)** | 120.3 (83.4) | 0.839 | 154.5 (102.1) | 0.064 | 204.3 (225.1) | 0.638 | 131.0 (120.1) | 0.250 | 22.0 (29.25) | 1.000 |
| **IL-6**  **(%)** | 157.7 (67.0) | **0.006*** | 129.6 (104.2) | 0.733 | 40.1  (44.4) | 0.831 | 11.6  (24.3) | 0.648 | 214.9 (192.0) | 0.313 |
| **IL-8**  **(%)** | 37.4 (16.9) | **0.047*** | 16.6 (18.0) | 0.733 | 1.0  (22.5) | 0.365 | -16.1 (5.5) | **0.022*** | 8.0 (25.6) | 0.547 |
| **IL-10 (%)** | 58.6 (35.8) | 0.376 | 99.4 (44.7) | **0.021*** | 65.7 (28.8) | **0.046*** | 45.6  (37.3) | 0.250 | 169.7 (150.8) | 0.078 |
| **MIP-1β (%)** | 37.2 (13.9) | **0.020*** | 17.1 (9.1) | 0.088 | 1.9 (12.4) | 0.882 | 7.5 (9.4) | 0.456 | 82.2 (43.3) | **0.023*** |
| **TNF**  **(%)** | 101.7 (89.8) | 0.685 | 51.2 (23.6) | 0.053 | 43.3 (39.1) | 0.294 | -13.4 (12.3) | 0.311 | 37.2 (32.6) | 0.461 |
| **VEGF (%)** | 1381.0 (902.8) | **0.043*** | 1610.0 (1232.0) | 0.160 | 366.7 (332.1) | 0.469 | 2839.0 (2833.0) | 0.563 | 2367.0 (1834.0) | 0.156 |

Data represented as means of percentage changes (SEM). *p-value indicates significant statistical difference between time point vs. baseline values as determined using one sample t-test or Wilcoxon Signed Rank test when values were not normally distributed.
